# Common Electrolyte and Metabolic Abnormalities Among Thyroid Patients

**DOI:** 10.7759/cureus.15338

**Published:** 2021-05-30

**Authors:** Hind A Alqahtani, Abdullateef A Almagsoodi, Nouf D Alshamrani, Tawfiq J Almalki, Abdulhadi M Sumaili

**Affiliations:** 1 Internal Medicine, Aseer Central Hospital, Abha, SAU

**Keywords:** thyroid disease, dysfunction, electrolyte disturbances, endocrine, metabolism, metabolic syndrome

## Abstract

The prevalence of thyroid diseases is high in the general population and causes serious abnormalities and disorders that can affect the quality of life. Many complications can result from poor or inappropriate management of the disease, leading to serious cardiovascular and metabolic complications. In the present review study, we aim to discuss the effect of thyroid diseases on metabolic and electrolyte abnormalities and the potential correlation with some common disorders. Evidence from previous studies has demonstrated that thyroid dysfunctions hugely affect the metabolism of glucose in the bodies of the affected patients, which can lead to the development of both type 1 and 2 diabetes mellitus (DM). Hyperthyroidism can lead to the development of impaired glucose tolerance and secondary diabetes mellitus. These include an increased glycogenolysis and gluconeogenesis process, increased intestinal absorption of glucose, and secondary ketogenesis and lipolysis, which will subsequently affect the functions of the insulin-secreting cells of the pancreas. Evidence showed that thyroid diseases are associated with the development of obesity and metabolic syndrome, and the management for these modalities should involve prior management of underlying thyroid diseases. Efforts should be made to adequately manage these cases with concomitant approaches to achieve the best clinical outcomes.

## Introduction and background

Thyroid dysfunction is mainly attributable to autoimmune pathologies. These include hypothyroidism, lymphocytic thyroiditis, Hashimoto’s thyroiditis, hyperthyroidism, or Grave’s disease [1-2]. The prevalence of thyroid disorders is high in the general population and causes various abnormalities and disorders that can affect the quality of life. Evidence shows that many factors such as the geographical distribution and ethnicity of individuals can affect the prevalence and development of thyroid disease. As previous investigations have previously reported that thyroid dysfunctions mainly occur among patients within the elderly population, it has been estimated that these disorders occur less frequently in children than in adults by 10 folds [3-5].

Primary hypothyroidism is most commonly caused by Hashimoto's thyroiditis. In patients with autoimmune thyroid diseases, the diagnosis is usually established when the patient reaches the age of 45-65 years. However, it has also been proven that dysfunction can appear in childhood. In addition, Hashimoto’s thyroiditis occurs more frequently in female than male individuals by 10-20 folds. Evidence also shows that the family history of the disease might be attributable to 50% of the underlying etiologies, as it has been reported that thyroid inflammation in this pathology is usually hereditary [6-7]. The frequency to develop hypothyroidism is affected by advancement in age and gender. Female individuals usually develop the disease five times more frequently than males. Hypothyroidism can be categorized into overt and subclinical types. The overt type has a prevalence rate of 0.1%-2% while the prevalence of subclinical hypothyroidism can reach up to 15% among females [8].

The prevalence of hyperthyroidism has been found to affect 2%-3% of the general population. Grave’s disease is the most common form of hyperthyroidism, as it nearly accounts for 75% of hyperthyroidism. Similar to hypothyroidism, evidence also shows that hyperthyroidism affects females more than males by 10 times the difference in the prevalence rates for each gender [8-9], with most patients diagnosed at the age of 48 [9-10]. Many complications can result from the poor or inappropriate management of the disease leading to serious cardiovascular and metabolic complications [9]. Hypothyroidism can also impact the different metabolic functions of the body, leading to a variety of clinical pictures and symptoms that can cause serious complications, which may be life-threatening [11]. In the present review study, we aim to discuss more on the effect of thyroid diseases on metabolic and electrolyte abnormalities and the potential correlation with some common disorders.

## Review

Electrolytes

Electrolytes play an important role in the body with regards to fluid balance, nerve conduction, and muscle contractions [12]. Thyroid hormones are considered a central regulatory system in maintaining a wide array of hemodynamic, thermodynamic, and metabolic functions. Furthermore, both thyroid and parathyroid disorders have been long established to affect serum calcium and sodium levels in the body [12]. Bharti et al. found in their study that there was a significant decrease in calcium in hypothyroidism and subclinical hypothyroidism patients [12]. This is mainly due to the effect of decreased levels of thyroxin. Thyroxin normally regulates calcium by releasing calcium ions from the cells. In addition, they reported significantly high phosphorus levels in hypothyroidism and subclinical hypothyroidism as well [12]. Furthermore, the same study revealed high levels of magnesium in hypothyroidism and subclinical hypothyroidism groups. Lastly, the sodium levels found in hypothyroidism and subclinical hypothyroidism patients were significantly lower than their control group while potassium levels did not have any changes in different groups [12]. This goes in line with what is in the literature except that sodium and potassium levels have been controversial among different studies and how thyroid-stimulating hormone (TSH), as well as T3 and T4, can have an effect on such levels [13]. Baajafer et al. previously reported that only 3.9% of their patients with hypothyroidism had hyponatremia [14]. Sun et al. also reported that hyponatremia was not a common finding among their population with extreme TSH elevations [15]. This was also supported by Wolf et al. who reported that the co-occurrence of hyponatremia with hypothyroidism is probably a coincidence and attributable to other causes [16]. Although chronic hypothyroidism was linked to a reduction in the levels of the antidiuretic hormone, inducing hyponatremia, recent evidence also supports these findings that causes other than hypothyroidism contribute to the development of hyponatremia, which can only occur in cases of severe hypothyroid disease and myxedema [17]. Previous investigations have also reported the prevalence of hypothyroidism among patients with hyponatremia. Nagata et al. reported that the prevalence of overt hypothyroidism was 1.3% among 71,817 patients with hyponatremia, and the severity of hyponatremia was significantly associated with the prevalence of hypothyroidism [18].

Type 1 and 2 diabetes mellitus (glucose abnormalities)

Evidence from previous studies has demonstrated that thyroid dysfunctions affect the metabolism of glucose in the bodies of the affected patients. Therefore, it can lead to the development of both type 1 and 2 diabetes mellitus (DM) [19]. Moreover, previous studies in the literature have demonstrated the potential co-existence of DM and autoimmune thyroid diseases, a correlation that can be frequently observed in children and young adults. Additionally, it has been previously estimated that the prevalence of hypothyroidism among children with type 1 DM is 3%-8%, and subclinical hypothyroidism can affect 5%-10% of the same population [20]. The prevalence of autoimmune thyroid disease is more common among female diabetics, as they usually develop subclinical hypothyroidism due to laboratory-observed high levels of TSH [19].

Hypoglycemia was evaluated in patients with Hashimoto's thyroiditis in a previous investigation by Gierach et al. [21]. The authors reported that among the population that suffered from Hashimoto’s thyroiditis in their study, nearly 28% suffered from a concomitant diagnosis with type 1 DM. Additionally, they reported that impaired glucose metabolism or tolerance was found in 17% of these patients. Accordingly, the authors suggested that glucose disturbances and type 1 DM can affect a huge proportion of patients with Hashimoto’s thyroiditis. Hyperthyroidism has been previously reported to correlate with the development of diabetes, being a risk factor. Among patients suffering from hyperthyroidism, it was previously reported that half of them develop impaired glucose tolerance and around 3% of these patients will eventually develop DM.

Many causes secondary to hyperthyroidism are attributable to the development of impaired glucose tolerance and secondary DM. These include the increased glycogenolysis and gluconeogenesis process, increased intestinal absorption of glucose, and secondary ketogenesis and lipolysis, which will subsequently affect the functions of the insulin-secreting cells of the pancreas [22]. Therefore, it is essential to care for the management of hyperthyroidism in these patients to enhance the metabolism of carbohydrates and reduce blood glucose levels [23]. As evidence shows that endocrinal comorbidities can co-exist with DM in less than one-third of the cases, it is recommended that a comprehensive analysis of patients with DM should be approached looking for any underlying thyroid abnormality, especially autoimmune thyroiditis [24-25].

Malabsorption disorders

Previous investigations have demonstrated a potential correlation between autoimmune thyroiditis and malabsorption disorders, represented mainly in celiac disease [26]. Celiac disease is an autoimmune disease that mainly affects the intestine by inducing serious damage and atrophy to the intestinal villae, leading to major digestion problems. The most frequent symptoms will include the development of a state of malabsorption leading to huge losses in electrolytes and nutrients. The chronicity of the condition will eventually lead to malnutrition, causing features such as anemia and loss of weight in addition to the reduced levels of electrolytes and blood proteins in the serum of the affected patient.

A previous investigation by Kawicka et al. also reported that in patients with autoimmune thyroiditis, developing celiac disease can be more common in these patients than the normal population by up to 13 folds increase in the risk [27]. Another investigation by Larizza et al. also reported that the prevalence of celiac disease increased in patients with autoimmune thyroiditis by 7.7% in their young children and adolescent population [28]. Overall, celiac disease has been previously used as a model by many investigations for the assessment and study of many autoimmune diseases [29]. Moreover, evidence shows that celiac disease can be a major risk factor for the development of many other autoimmune diseases and can also lead to the exacerbation of the symptoms of other autoimmune disorders [30].

Negligence and delayed diagnosis of celiac disease might complicate the case and lead to the development of other autoimmune disorders. Therefore, it should be recommended that all patients that suffer from autoimmune thyroid diseases should be subjected to a regular examination for celiac disease. A previous multicentre investigation by Ansaldi et al. aimed to find a potential correlation between celiac disease and autoimmune thyroid diseases [30]. The authors reported that among 26.2% of patients that suffered from celiac disease in their pediatric population, autoimmune thyroid disease was found as the second comorbidity for these patients. Therefore, it is essential to assess for both modalities as the development of either of them can eventually affect the pathophysiology and prognosis of the other.

Previous studies have also demonstrated the co-existence of celiac disease and autoimmune thyroiditis, in addition to the development of diabetes among the adult population. Accordingly, it has been suggested that the proper management of celiac disease by providing patients with gluten-free diets can effectively reduce the intensity and severity of the thyroid dysfunction symptoms that are associated with the disease. In another perspective, a previous review by Virili et al. indicated that the occurrence of malabsorption syndromes can significantly impact the levels of T4, which can cause secondary hypothyroidism [31]. Eventually, it is recommended that adequate screening for both modalities should be offered whenever the diagnosis of either of them was suspected [32-33].

Metabolic syndrome and obesity

Many risk factors and etiologies have been stratified for developing obesity. Among these, environmental, genetic, and endocrinal factors are marked as the main causes that attribute to the development of obesity [34], as it is widely known that thyroid hormones contribute to nearly 30% of energy expenditure. These hormones play vital roles in the regulation of the metabolic pathways and have a major role in the rough endoplasmic reticulum on a cellular basis [35]. Imbalances between food intake and energy loss can lead to the development of obesity. Obesity can influence thyroid hormone levels and can also lead to a permanent effect on them that usually normalizes following weight loss [36-37].

**Figure 1:**
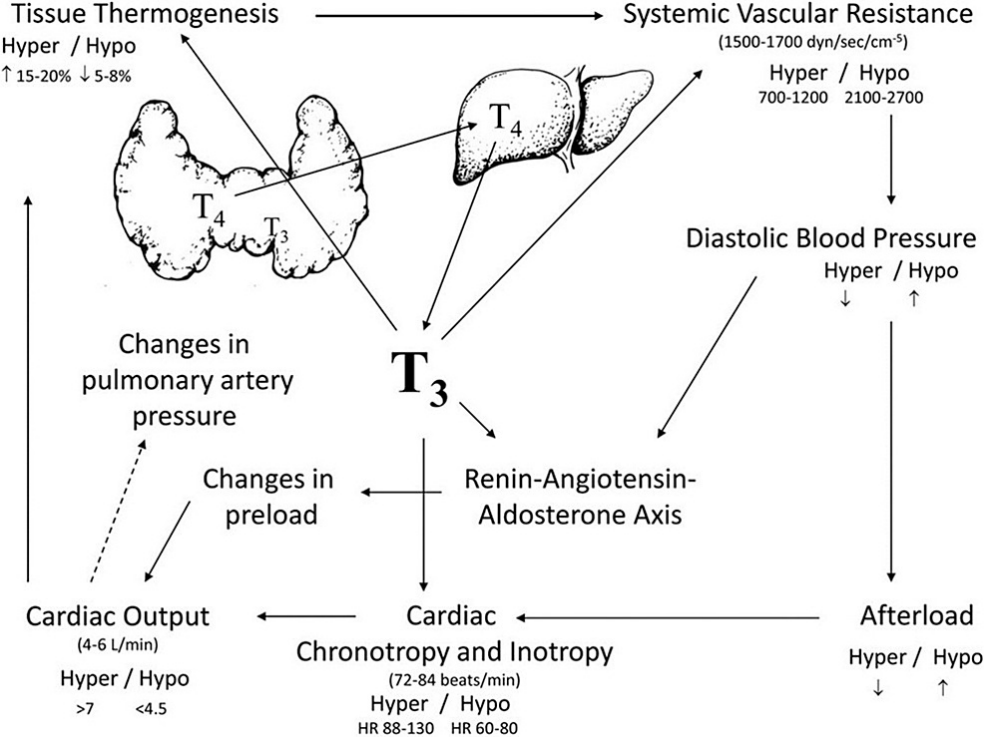
Effects of thyroid hormone on the hemodynamics of the cardiovascular system

Reduction in metabolic activities and thermal regulation leading to increased weight gain and obesity are usually consequences of hypothyroidism. Previous investigations have referred to a potential association between subclinical hypothyroidism and weight gain, through the potential effects on lipid metabolism, which can eventually increase the risk of cardiovascular diseases as well (Figure 1) [38-39]. On the other hand, patients with hyperthyroidism usually suffer from weight loss, although it has been frequently reported that these patients usually have increased appetite and energy production [40]. Hashimoto’s thyroiditis has also been previously linked to having weight disturbances and other gastrointestinal manifestations [41-42]. A previous investigation stated that enhancing the quality and quantity of dietary intake can significantly enhance gastrointestinal symptoms like constipation in female patients suffering from Hashimoto’s thyroiditis and obesity [42-43]. The effect of hypothyroidism can also extend to many metabolic and biological processes within the body. It is now well-known that reduced thyroid hormones can significantly increase blood pressure, worsen the lipid profile, and increase the levels of many harmful parameters and inflammatory cytokines as C-reactive protein and homocysteine, which may cause multiple toxic processes. Atherosclerosis has been previously reported to be correlated with hypothyroidism [44].

A previous investigation by Ruhla et al. previously reported that metabolic syndrome is associated with an estimated range for TSH levels of 0.3-4.5 mU/L [45]. Moreover, a slight elevation in the TSH levels at the upper limit of the normal levels of the hormone was associated with more obesity and more frequently elevated triglycerides levels in the serum of these patients. Evidence also showed that to maintain acceptable lipid profiles, TSH levels should not exceed 2.5 mU/L. A previous investigation by Dunats and Wartofsky also reported that when the TSH levels were above 10 mU/L, significant changes in the metabolism of cholesterol and lipids were noticed in these patients with subclinical hypothyroidism [46]. Accordingly, it has been declared that the risk of cardiovascular diseases significantly increases in correlation with such events, which are mainly attributable to thyroid dysfunction.

Previous investigations have also reported that body mass index (BMI) can be used for predicting the levels of TSH in obese patients since a solid correlation has been evidenced between obesity and thyroid hormones. Interestingly, a previous investigation by Iacobellis et al. reported that the metabolism of the adipose tissue can be directly or indirectly correlated with noticed activities of the thyroid gland [47]. It is worth mentioning that the authors have also reported that the BMI and TSH levels were significantly correlated with each other. Besides, a previous study has reported that any decrease in the TSH levels within the normal range is significantly associated with a constant reduction in energy production in the affected patients [48]. Although thyroid hormones might have a direct link between obesity and weight loss, it should not be recommended that thyroid hormones be therapeutically prescribed for obese patients due to the unfavorable adverse events and complications that may be associated [39,49-50]. Moreover, dietary planning and follow-up should be considered when treating patients with thyroid dysfunctions as previously discussed in this review.

## Conclusions

In this study, we reviewed the potential effect of thyroid diseases on some common metabolic and electrolyte imbalance disorders. Evidence showed that thyroid diseases are associated with the development of obesity and metabolic syndrome, and the management of these modalities should involve the prior management of the underlying thyroid diseases. Moreover, diabetes mellitus is also linked to thyroid diseases, whether as autoimmune comorbidity or secondary to impaired glucose tolerance. Malabsorption and celiac disease also constitute common disorders that can affect many other metabolic and electrolyte parameters and have been reported to be common with thyroid dysfunction. Efforts should be offered to adequately manage these cases with concomitant approaches to achieve the best clinical outcomes.

## References

[1] Swain M, Swain T, Mohanty BK (2005). Autoimmune thyroid disorders—an update. Indian J Clin Biochem.

[2] Mincer DL, Jialal I (2020). Hashimoto Thyroiditis. https://www.ncbi.nlm.nih.gov/books/NBK459262/.

[3] McGrogan A, Seaman HE, Wright JW, de Vries CS (2008). The incidence of autoimmune thyroid disease: a systematic review of the literature. Clin Endocrinol (Oxf).

[4] Galofré JC, García-Mayor RV, Fluiters E, Fernàndez-Calvet L, Rego A, Pàramo C, Andrade MA (1994). Incidence of different forms of thyroid dysfunction and its degrees in an iodine sufficient area. Thyroidology.

[5] Lai X, Xia Y, Zhang B, Li J, Jiang Y (2017). A meta-analysis of Hashimoto's thyroiditis and papillary thyroid carcinoma risk. Oncotarget.

[6] Jankovic B, Le KT, Hershman JM (2013). Hashimoto's thyroiditis and papillary thyroid carcinoma: is there a correlation?. J Clin Endocrinol Metab.

[7] Devdhar M, Ousman YH, Burman KD (2007). Hypothyroidism. Endocrinol Metab Clin North Am.

[8] De Leo S, Lee SY, Braverman LE (2016). Hyperthyroidism. Lancet.

[9] McDermott MT (2020). Hyperthyroidism. Ann Intern Med.

[10] Tunbridge WM, Evered DC, Hall R (1977). The spectrum of thyroid disease in a community: the Whickham survey. Clin Endocrinol (Oxf).

[11] Gessl A, Lemmens-Gruber R, Kautzky-Willer A (2012). Thyroid disorders. Handb Exp Pharmacol.

[12] Bharti A, Shrestha S, Rai R, Singh MK (2015). Assessment of serum minerals and electrolytes in thyroid patients. Int J Adv Sci Res.

[13] Schwarz C, Leichtle AB, Arampatzis S, Fiedler GM, Zimmermann H, Exadaktylos AK, Lindner G (2012). Thyroid function and serum electrolytes: does an association really exist?. Swiss Med Wkly.

[14] Baajafer FS, Hammami MM, Mohamed GE (1999). Prevalence and severity of hyponatremia and hypercreatininemia in short-term uncomplicated hypothyroidism. J Endocrinol Invest.

[15] Sun GE, Pantalone KM, Hatipoglu B (2012). Hypothyroidism as a cause of hyponatremia: fact or fiction?. Endocr Pract.

[16] Wolf P, Beiglböck H, Smaijs S (2017). Hypothyroidism and hyponatremia: rather coincidence than causality. Thyroid.

[17] Liamis G, Filippatos TD, Liontos A, Elisaf MS (2017). Management of endocrine disease: hypothyroidism-associated hyponatremia: mechanisms, implications and treatment. Eur J Endocrinol.

[18] Nagata T, Nakajima S, Fujiya A, Sobajima H, Yamaguchi M (2018). Prevalence of hypothyroidism in patients with hyponatremia: a retrospective cross-sectional study. PLoS One.

[19] Holl RW, Bohm B, Loos U, Grabert M, Heinze E, Homoki J (1999). Thyroid autoimmunity in children and adolescents with type 1 diabetes mellitus. Effect of age, gender and HLA type. Horm Res.

[20] Mantovani RM, Mantovani LM, Dias VM (2007). Thyroid autoimmunity in children and adolescents with type 1 diabetes mellitus: prevalence and risk factors. J Pediatr Endocrinol Metab.

[21] Gierach M, Gierach J, Skowrońska A, Rutkowska E, Spychalska M, Pujanek M, Junik R (2012). Hashimoto’s thyroiditis and carbohydrate metabolism disorders in patients hospitalised in the department of endocrinology and diabetology of Ludwik Rydygier Collegium Medicum in Bydgoszcz between 2001 and 2010. Endokrynologia Polska.

[22] Biondi B, Kahaly GJ, Robertson RP (2019). Thyroid dysfunction and diabetes mellitus: two closely associated disorders. Endocr Rev.

[23] Hage M, Zantout MS, Azar ST (2011). Thyroid disorders and diabetes mellitus. J Thyroid Res.

[24] Siwamogsatham O, Alvarez JA, Tangpricha V (2014). Diagnosis and treatment of endocrine comorbidities in patients with cystic fibrosis. Curr Opin Endocrinol Diabetes Obes.

[25] Krysiak R, Rudzki H, Okopień B (2012). Diabetes and prediabetes in endocrine disorders [Article in Polish]. Wiad Lek.

[26] Selimoğlu MA, Ertekin V (2005). Autoimmune thyroid disease in children with celiac disease. J Pediatr Gastroenterol Nutr.

[27] Kawicka A, Regulska-Ilow B, Regulska-Ilow B (2015). Metabolic disorders and nutritional status in autoimmune thyroid diseases. Postepy Hig Med Dosw (Online).

[28] Larizza D, Calcaterra V, De Giacomo C (2001). Celiac disease in children with autoimmune thyroid disease. J Pediatr.

[29] López Casado MÁ, Lorite P, Ponce de León C, Palomeque T, Torres MI (2018). Celiac disease autoimmunity. Arch Immunol Ther Exp (Warsz).

[30] Ansaldi N, Palmas T, Corrias A (2003). Autoimmune thyroid disease and celiac disease in children. J Pediatr Gastroenterol Nutr.

[31] Virili C, Antonelli A, Santaguida MG, Benvenga S, Centanni M (2019). Gastrointestinal malabsorption of thyroxine. Endocr Rev.

[32] Collin P, Kaukinen K, Välimäki M, Salmi J (2002). Endocrinological disorders and celiac disease. Endocr Rev.

[33] Sategna-Guidetti C, Volta U, Ciacci C (2001). Prevalence of thyroid disorders in untreated adult celiac disease patients and effect of gluten withdrawal: an Italian multicenter study. Am J Gastroenterol.

[34] Tappia PS, Ramjiawan B, Dhalla NS (2020). Pathophysiology of Obesity-induced Health Complications. https://link.springer.com/content/pdf/10.1007/978-3-030-35358-2.pdf.

[35] Bandurska-Stankiewicz E (2013). Thyroid hormones - obesity and metabolic syndrome. Thyroid Res.

[36] Hill JO, Wyatt HR, Peters JC (2012). Energy balance and obesity. Circulation.

[37] Spiegelman BM, Flier JS (2001). Obesity and the regulation of energy balance. Cell.

[38] Sami A, Iftekhar MF, Rauf MA, Sher A (2018). Subclinical hypothyroidism among local adult obese population. Pak J Med Sci.

[39] Rodondi N, Newman AB, Vittinghoff E, de Rekeneire N, Satterfield S, Harris TB, Bauer DC (2005). Subclinical hypothyroidism and the risk of heart failure, other cardiovascular events, and death. Arch Intern Med.

[40] Reinehr T (2010). Obesity and thyroid function. Mol Cell Endocrinol.

[41] Basińska MA, Merc M, Juraniec O (2009). Mood of individuals with Graves-Basedow's disease and Hashimoto's disease. Endokrynol Pol.

[42] Baranowska-Bik A, Bik W (2020). The association of obesity with autoimmune thyroiditis and thyroid function-possible mechanisms of bilateral interaction. Int J Endocrinol.

[43] Song RH, Wang B, Yao QM, Li Q, Jia X, Zhang JA (2019). The impact of obesity on thyroid autoimmunity and dysfunction: a systematic review and meta-analysis. Front Immunol.

[44] Ichiki T (2010). Thyroid hormone and atherosclerosis. Vascul Pharmacol.

[45] Ruhla S, Weickert MO, Arafat AM (2010). A high normal TSH is associated with the metabolic syndrome. Clin Endocrinol (Oxf).

[46] Duntas LH, Wartofsky L (2007). Cardiovascular risk and subclinical hypothyroidism: focus on lipids and new emerging risk factors. What is the evidence?. Thyroid.

[47] Iacobellis G, Ribaudo MC, Zappaterreno A, Iannucci CV, Leonetti F (2005). Relationship of thyroid function with body mass index, leptin, insulin sensitivity and adiponectin in euthyroid obese women. Clin Endocrinol (Oxf).

[48] Al-Adsani H, Hoffer LJ, Silva JE (1997). Resting energy expenditure is sensitive to small dose changes in patients on chronic thyroid hormone replacement. J Clin Endocrinol Metab.

[49] Liu G, Liang L, Bray GA (2017). Thyroid hormones and changes in body weight and metabolic parameters in response to weight loss diets: the POUNDS LOST trial. Int J Obes (Lond).

[50] Danzi S, Klein I (2014). Thyroid disease and the cardiovascular system. Endocrinol Metab Clin North Am.

